# Active electrode location does not influence weight gain after subthalamic nucleus deep brain stimulation surgery in Parkinson disease

**DOI:** 10.1007/s00702-026-03201-4

**Published:** 2026-06-13

**Authors:** Amelia Heston, Asra Askari, Kara Wyant, Parag G. Patil, Kelvin L. Chou, Daniel Leventhal

**Affiliations:** 1 University of Michigan Neurology, Ann Arbor, USA; 2 VA Ann Arbor Healthcare System Neurology, Ann Arbor, USA; 3 University of Michigan Neurosurgery, Ann Arbor, USA; 4 Biomedical Engineering, University of Michigan, Ann Arbor, USA

**Keywords:** Parkinson disease, Weight gain, Deep brain stimulation, Subthalamic nucleus

## Abstract

Weight change (WC) is a well-recognized and poorly understood side effect of deep brain stimulation (DBS). The aim of our study was to determine the relationship between active electrode location in the subthalamic nucleus (STN) and post-DBS WC. This was a single center retrospective study of 82 patients who underwent STN DBS. Two weeks after surgery, a head CT was obtained and co-registered to the patient’s preoperative 3T MRI. The STN midpoint was identified, and the location of the active electrode was defined with respect to the STN midpoint in Talairach space. Least squares regression models were applied to evaluate the role of active electrode location on WC, as well as the relationships between WC and the explanatory variables sex, age, disease duration, change in levodopa equivalent dose (LED), change in MDS-UPDRS III scores, and change in dyskinesias before and after DBS. Active electrode contact location was not significantly associated with WC along any direction in either hemisphere. WC was significantly associated with change in dyskinesias (slope = -1.8 lbs/point, R^2^ = 0.072, *p* = 0.025), but only in a univariate model. Our findings suggest that active electrode contact location does not influence WC after STN DBS.

## Introduction

Deep brain stimulation (DBS) is an effective treatment for the motor symptoms of Parkinson disease (PD) (Deuschl et al. 2006; Weaver et al. 2009, 2012) but has non-motor side effects including post-procedural weight change (WC), usually weight gain (Barichella et al. 2003; Gironell et al. 2002; Tuite et al. 2005; Strowd et al. 2010; Macia et al. 2004). The etiology of WC after DBS is poorly understood. Proposed mechanisms include reduction in dyskinesia, decreased energy expenditure, reduction in levodopa equivalent dosing (LED) and associated gastrointestinal side effects, hormonal factors, change in intake, and stimulation of structures regulating reward and energy balance (Barichella et al. 2003; Montaurier et al. 2007; Aiello et al. 2017; Steinhardt et al. 2022; Balestrino et al. 2017).

Subthalamic nucleus (STN) stimulation is associated with greater postoperative WC than globus pallidus internus (GPi) stimulation in most studies (Monteferrante et al. 2020; Mills et al. 2012; Sauleau et al. 2009; Eisinger et al. 2023), though one study found no significant difference (Locke et al. 2011). Suggested anatomical subdivisions of the STN include a dorsolateral sensorimotor area, a ventromedial associative area, and a medial limbic area (Benarroch 2008; Parent and Hazrati 1995). Improvement in PD motor symptoms after STN DBS has been attributed to stimulation of the dorsolateral sensorimotor area (Herzog et al. 2004; Voges et al. 2002). Non-motor side effects of STN DBS have also been correlated with active electrode contact location within the STN (Greif et al. 2021; Mossner et al. 2020). A specific electrode location within the STN may explain WC after DBS.

The relationship between active electrode contact localization and WC has been explored (Balestrino et al. 2017; Eguchi et al. 2021; Růžička et al. 2012; Steinhardt et al. 2023). Existing data is inconsistent, with multiple electrode locations having been associated with post-DBS WC. The aim of our study is to clarify the potential relationship between DBS electrode contact location in relation to the STN and post-DBS WC.

## Methods

### Clinical data

**Fig. 1:**
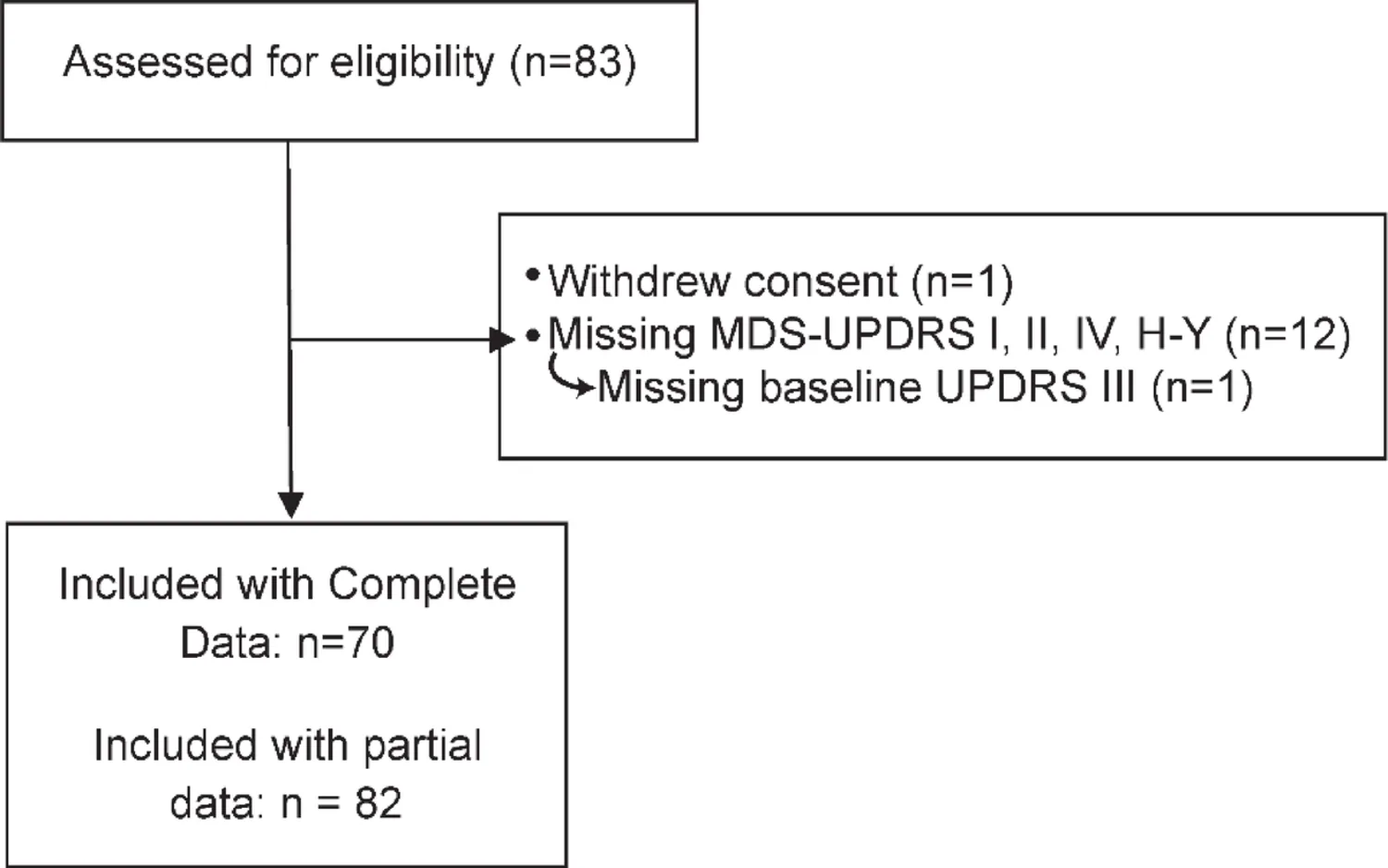
CONSORT style flow chart of patients included/excluded from the analysis

This is a retrospective study of 82 patients who underwent STN DBS in the University of Michigan Surgical Therapies Improving Movement (STIM) program between 2009 and 2019. All patients that underwent DBS and consented to participate in the research database are included in this analysis (Fig. 1). All had a diagnosis of PD with motor fluctuations despite optimal medical management or disabling tremor unresponsive to levodopa. Patients were ineligible for DBS if they had structural abnormalities on preoperative MRI preventing accurate placement of a DBS lead in the STN or dementia by neuropsychological testing. More details regarding the selection criteria to undergo DBS have been described previously (Chou et al. 2013; Houshmand et al. 2014). Informed consent was obtained from all patients in accordance with the policies of the Medical Institutional Review Board of the University of Michigan.

Patients analyzed in this study had their weight measured pre-operatively as well as between 162 and 701 days after DBS surgery. Reported weights are routine clinic measurements; we did not prospectively define a protocol for weight measurement (e.g., clothing status, fasting). All patients also had adequate imaging that allowed for localization of the active electrode contact. Additional clinical data used for this analysis included sex, age, disease duration, Movement Disorder Society revision of the Unified Parkinson Disease Rating Scale (MDS-UPDRS parts I-IV), Hoehn and Yahr stage (HY), and LED. The first 12 patients enrolled were evaluated with the UPDRS. For those patients, UPDRS III scores were converted to MDS-UPDRS III scores by adding 7 (Hentz et al. 2015). These 12 patients were excluded from analyses of MDS-UPDRS parts I, II, or IV. One patient did not have baseline UPDRS III or MDS-UPDRS III scores and was excluded from the regression between weight change and change in MDS-UPDRS III scores. The number of patients with data for each variable is summarized in Table 1. LED was calculated as in Tomlinson et al. 2010 (Tomlinson et al. 2010). BMI was calculated as weight in kg divided by height in meters squared. BMI classifications were assigned as defined by the World Health Organization (underweight: < 18.5 kg/m^2^, healthy: 18.5–25 kg/m^2^, overweight: 25–30 kg/m^2^, obese class 1: 30–35 kg/m^2^, obese class 2: 35–40 kg/m^2^, obese class 3: > 40 kg/m^2^).

### Active electrode contact localization

The surgical procedure for STN DBS at our institution has been described previously (Mossner et al. 2020; Chou et al. 2013). Briefly, there were two stages for bilateral DBS STN surgery. During the first stage, one quadripolar electrode (Medtronic 3389 DBS lead, Minneapolis, MN) was implanted into each STN and microelectrode recordings were used to map the borders. The DBS lead was placed under fluoroscopic guidance with the tip of the lead placed near the ventral border of the STN. The second stage was performed 2–4 weeks later at which time the implantable pulse generator was connected to the leads and associated extension wires.

Two weeks after surgery, a head CT was obtained and co-registered to the patient’s preoperative 3 T MRI (Analyze, AnalyzeDirect, Inc., Overland Park, KS). Details regarding our imaging protocol have been described previously (Patil et al. 2012). The STN midpoint was visualized and defined as the point midway between the STN rostral and caudal pole. The location of the active electrode contact was defined as the distance to the STN midpoint in Talairach space, yielding six variables: X (medial-lateral), Y (posterior-anterior), and Z (ventral-dorsal) axes in the left and right STN. Anterior, dorsal, and lateral directions are positive.

### Statistical analysis

Regression models were fit to assess the relationship between WC and the explanatory variables sex, age, disease duration, change in LED, time to follow-up, change in UPDRS III scores, and change in MDS-UPDRS IV dyskinesia scores. The dyskinesia score was taken as the sum of MDS-UPDRS IV.1 (time spent with dyskinesias) and IV.2 (functional impact of dyskinesias) scores. We performed regression using the Huber loss to account for outliers (δ = 1.345, the Matlab default; other values of δ did not meaningfully change the results) and stepwise regression (forward selection followed by backwards elimination) for robustness. We considered p-values significant at α = 0.05 for the stepwise model. The stepwise model (Matlab stepwiselm function using the “SSE” criterion) started with a constant model and added predictors one at a time if they significantly reduced the sum of squared errors (*p* < 0.05). Once all viable terms had been added, they were removed if *p* > 0.10 for the change in sum of squared errors with its removal. Because data were only available for 70 patients for the full model, we repeated these analyses without the change in dyskinesia score as a predictive factor, allowing us to include 11 more patients.

We also applied regression using the Huber loss to evaluate the role of electrode active contact location on WC at the first follow-up score. P-values for the slopes were considered significant at α = 0.05/6 = 0.0083 (Bonferroni correction). Power was estimated for the least squares model using the pwr.f2.test function in R (version 4.5.1). We repeated all regressions against active contact location with the addition of quadratic terms to test for nonlinearities. None of the quadratic terms passed significance, arguing against a nonlinear relationship between WC and contact location. Finally, active contact locations were transformed along their principal components, and the regressions repeated to determine if contact location along the principal component axes predicted WC.

All calculations were performed using Matlab R2026a unless otherwise specified.

## Results

### Baseline characteristics

**Table 1:** Baseline and follow-up characteristics of the patient cohort

Characteristic	Preoperative - Mean (SD)	Postoperative - Mean (SD)	Number of patients with data
Sex (M/F)	58/24	**-**	82
HY (stage 0/1/2/3/4/5)	0/1/45/14/8/2	**-**	70
Age at Initial Evaluation (years)	62.8 (7.5)	**-**	82
Disease Duration (years)	9.8 (5.0)	**-**	82
MDS UPDRS I	14.4 (5.4)	11.7 (6.0)	70
MDS UPDRS II	17.6 (7.2)	14.1 (7.0)	70
MDS UPDRS IV	9.8 (4.1)	3.2 (3.3)	70
MDS UPDRS III on medication	21.1 (11.9)	35.4 (14.9) off stim, 19.7 (10.7) on stim	81 pre-op 82 post-op
MDS UPDRS III off medication	42.2 (15.1)	47.4 (16.3) off stim, 28.1 (13.4) on stim	81 pre-op 82 post-op
LED (mg)	1449.5 (694.8)	726.8 (543.7)	82
Weight (lbs/kg)	176.6 (42.7)/80.1 (19.4)	189.9 (44.1)/86.1 (20.0)	82
BMI (kg/m^2^)	26.7 (5.4)	28.8 (5.7)	82
WHO BMI class (underweight/healthy/overweight/obese class 1/2/3)	4/26/36/9/6/1	3/17/35/16/7/4	82

Patient characteristics, descriptive statistics, and stimulation parameters are provided in Tables 1 and 2. Mean time to follow-up from stage I surgery was 294 +/- 106 days (mean +/- SD, minimum 162 days, maximum 701 days; median 255 days, interquartile range 227 to 318 days). WC did not vary significantly as a function of time to follow-up (linear regression, slope = 0.018 lbs/day, R^2^ = 0.021, *p* = 0.20). The patients had a mean baseline weight of 176.6 ± 42.7 pounds (80.1 +/- 19.4 kg) and a mean weight of 189.9 ± 44.1 pounds (86.1 +/- 20.0 kg) after DBS (*p* = 4.6 × 10^− 14^, paired t-test).

**Table 2:** DBS stimulation settings at follow-up

Stimulation Configuration	# of Patients	Pulse Width	# of Patients	Frequency	# of Patients	Voltage – mean (SD)
L STN DBS						
Monopolar	61	60	75	130	58	2.72 (0.59)
Bipolar	10	90	5	185	13	
Interleaved	9	Unknown	1	125	9	
Double Monopolar	1	Off	1	132	1	
Off	1			Off	1	
R STN DBS						
Monopolar	62	60	78	130	57	2.72 (0.66)
Bipolar	11	90	4	185	15	
Interleaved	7			125	9	
Double Monopolar	2			132	1	

### Effect of DBS on WC

71 of the 82 patients gained weight (WC > 0), and 11 experienced weight loss (WC < 0). The baseline weight for males undergoing DBS was 47.0 (21.4 kg) pounds more than females undergoing DBS (p *=* 1.4 × 10^− 6^, t-test) but WC after DBS was no different between males and females. Of 26 patients with a healthy BMI at baseline, 10 (38%) were classified as overweight or obese after DBS.

**Fig. 2:**
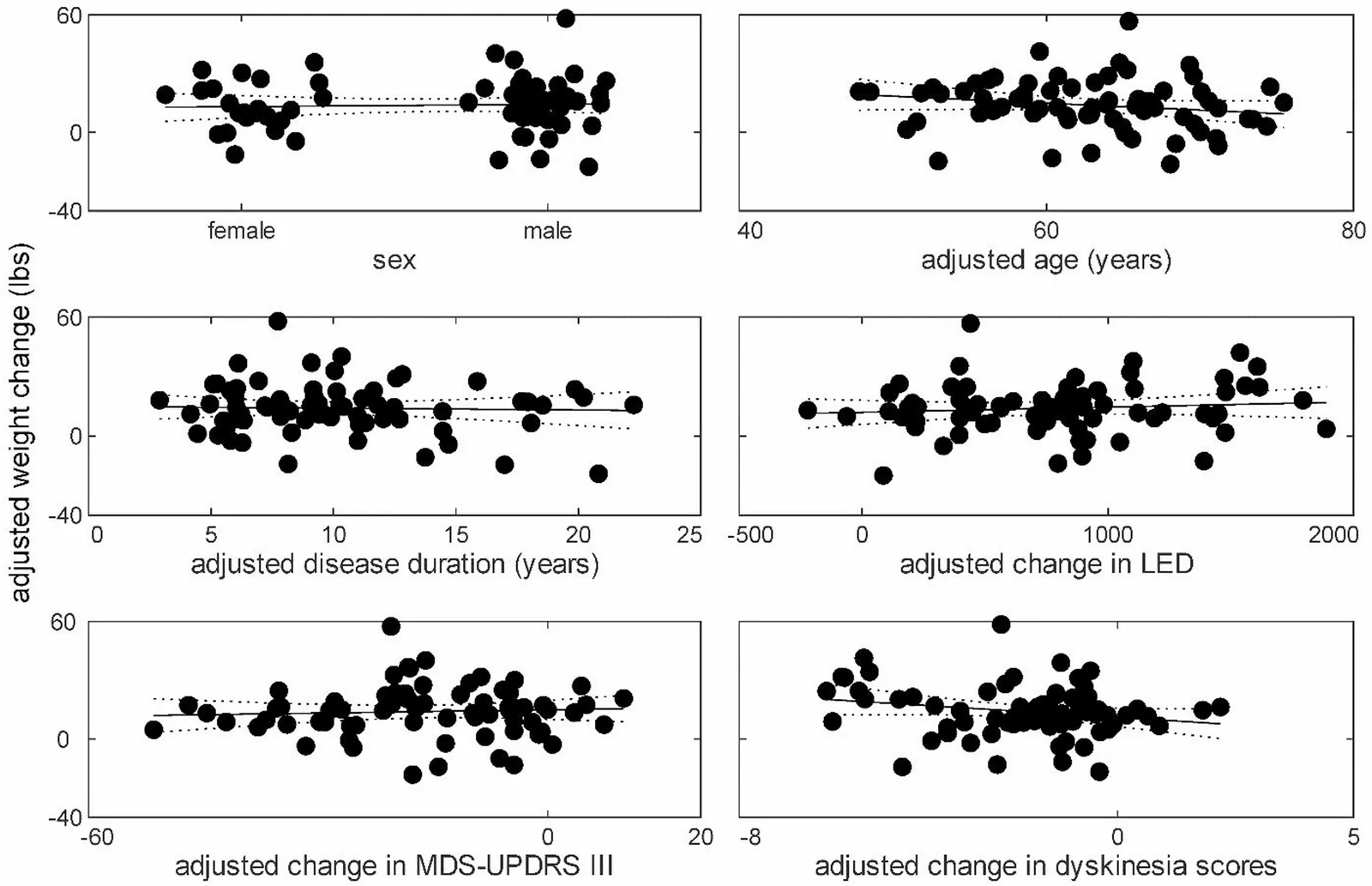
Added variable plots (regression lines for one variable adjusting for the effects of the others) for the regression of WC against sex, age, disease duration, change in LED, change in MDS-UPDRS III, and change in dyskinesia scores. Solid lines show the best fit, dotted lines show 95% confidence bounds. For the full model, adjusted R^2^ = 0.062, *p* = 0.12. Statistics for individual factors are given in Table 3

In the full regression model of WC as a function of sex, age, disease duration, change in LED, change in MDS-UPDRS, and change in dyskinesia score, none of the predictors were significant (Fig. 2; Table 3). In the stepwise model, only the change dyskinesia score was significantly predictive of WC (slope = −1.8 × 10^− 3^ lbs/levodopa equivalent, R^2^ = 0.072, *p* = 0.025). We repeated the analysis excluding the change in dyskinesia scores, which added 11 patients to the analysis. None of these factors significantly predicted WC with all predictors included. When predictors were added stepwise to the model, change in LED was predictive of WC (slope = 5.4 × 10^− 3^, R^2^ = 0.052, *p* = 0.040).

**Table 3:** Statistics for regression of WC against sex, age, disease duration, change in LED, change in MDS-UPDRS III (pre-op: off meds, post-op: off meds, on DBS), and change in dyskinesia score. The regression was repeated without the dyskinesia score, which added 11 patients to the model

variable	Full model (*n* = 70)		Model without dyskinesia score (*n* = 81)	
	slope	*p*	slope	*p*
sex	1.2	0.72	−1.3	0.69
age	−0.36 lbs/year	0.14	−0.29 lbs/year	0.16
disease duration	−0.090 lbs/year	0.80	−0.15 lbs/year	0.63
Δ LED	0.0026 lbs/ldopa equivalent	0.42	4.7 × 10^− 3^ lbs/ldopa equivalent	0.10
change in MDS-UPDRS III	0.056 lbs/point	0.62	−0.036 lbs/point	0.70
change in dyskinesia score	−1.5 lbs/point	0.07	--	--

37 patients reduced or eliminated dopamine agonists (pramipexole, ropinirole, or rotigotine) while 45 patients did not reduce agonist dose or were not taking one preoperatively (10 of these 45 patients were taking a dopamine agonist but did not reduce the dose). Reducing or eliminating dopamine agonists did not affect WC (WC with agonist reduction = 13.1 +/- 12.8 lbs [6.0 +/- 5.8 kg]; WC without agonist reduction = 13.6 +/- 13.8 lbs [6.2 +/- 6.3 kg], *p* = 0.87, 2-sample t-test).

### Effect of active electrode contact location on WC

**Fig. 3:**
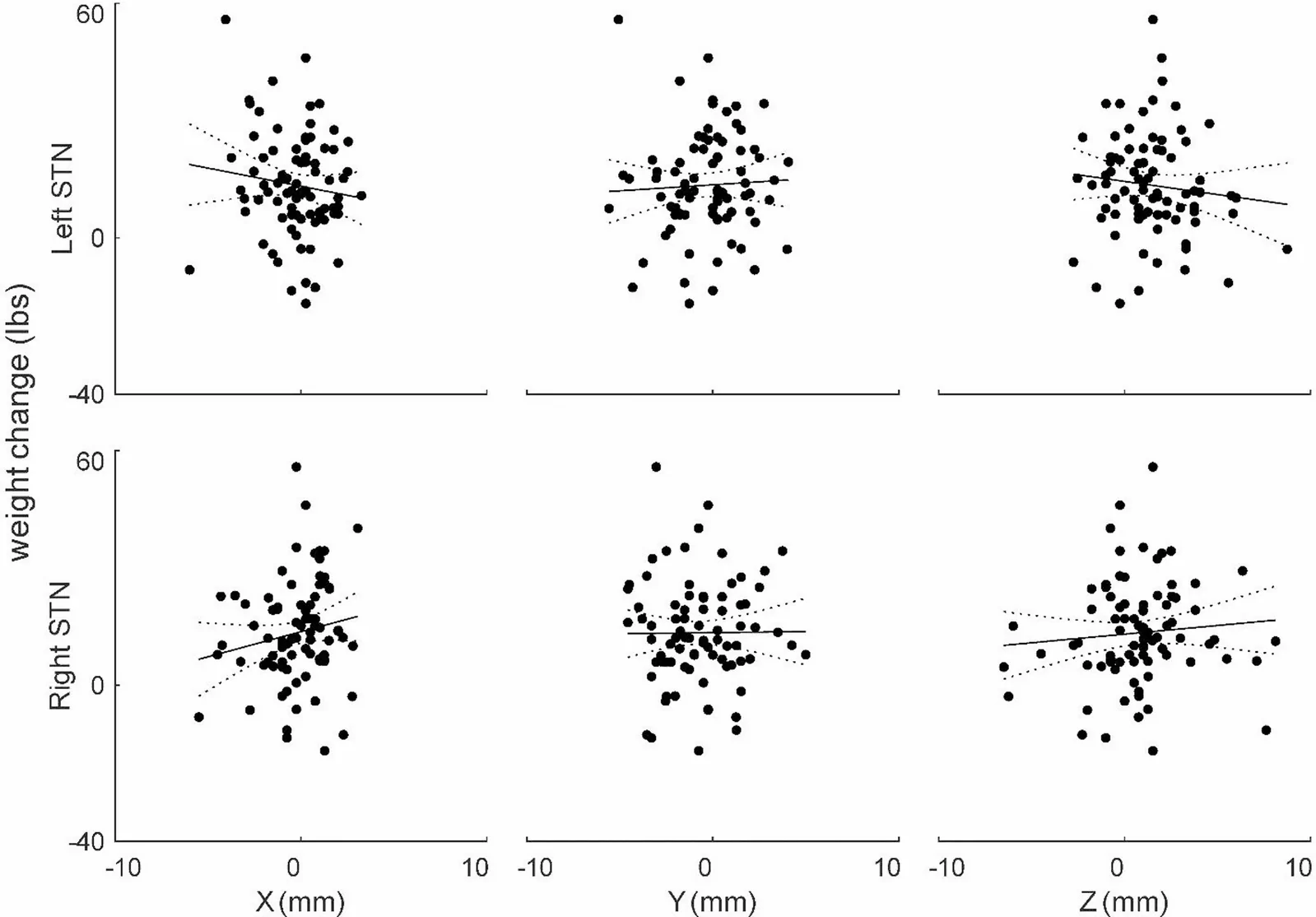
Relationships between WC and active contact position. None of the regressions achieved statistical significance. Solid lines show the best fit, dotted lines show 95% confidence bounds. Regression statistics are given in Table 4

**Fig. 4:**
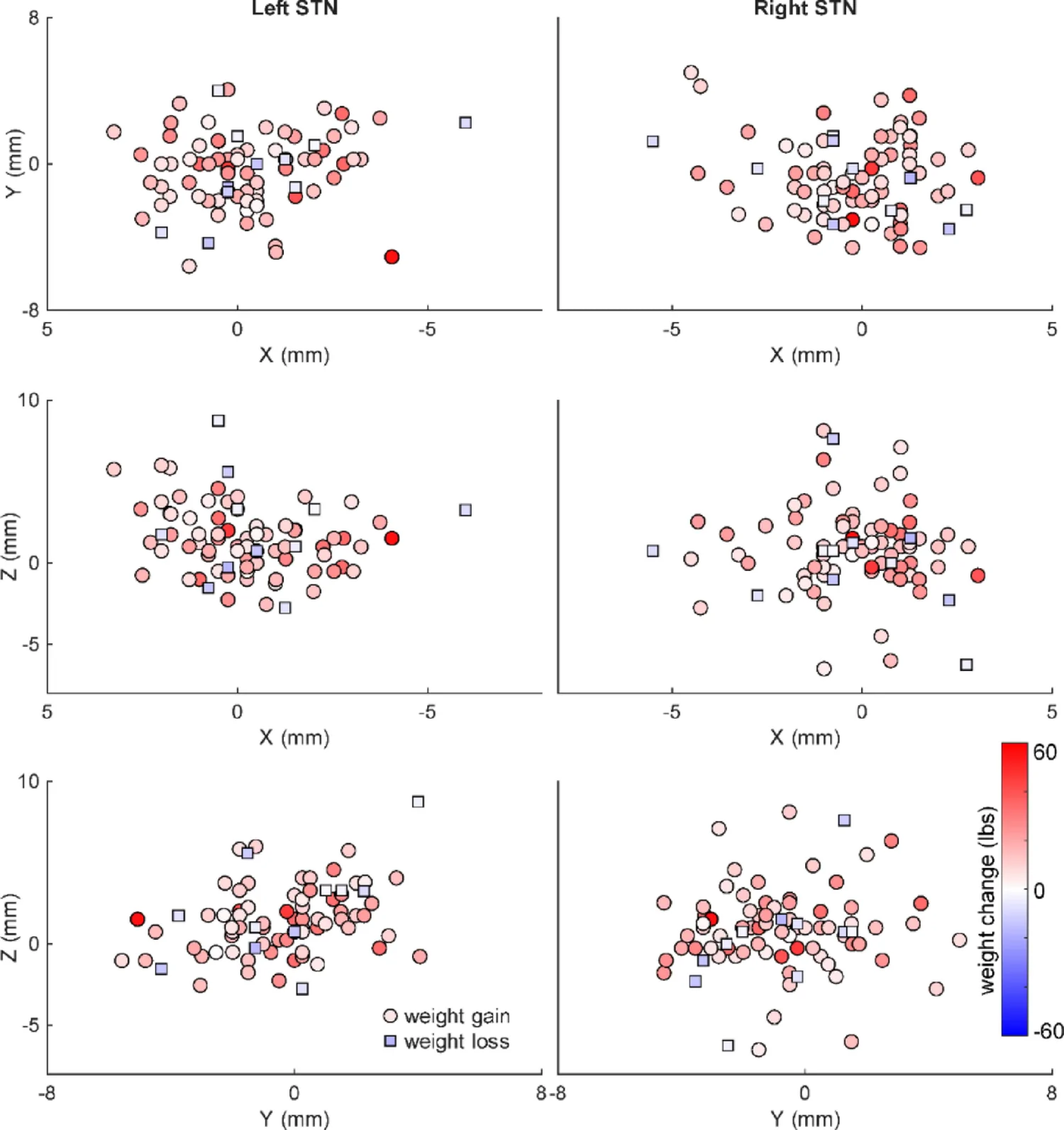
2-D scatter plots of active contact locations in each plane (X-Y, X-Z, Y-Z). Marker shape indicates the direction of WC (squares for weight loss, circles for weight gain). Shading intensity indicates the magnitude of the change

Active electrode contact locations in the medial-lateral, dorsal-ventral, and anterior-posterior axes in the left STN ranged from − 6.00 to 3.25, −5.58 to 4.06, and − 2.75 to 8.75 mm, respectively. In the right STN, they ranged from − 5.50 to 3.04, −4.57 to 5.25, and − 6.50 to 8.12 mm, respectively. Active electrode contact location was not significantly associated with WC along any direction in either hemisphere (Table 4; Figs. 3 and 4). We repeated all regressions with an additional quadratic term to test for nonlinearity – none of the quadratic terms were significant. Finally, we examined the possibility that position along the direction of greatest variance in contact location might account for WC. We performed PCA of the active contact locations, and regressed WC against active contact location along each principal component. Principal components for left STN active contacts explained 50.3%, 32.4%, and 17.4% of the variance in contact location; for right STN those values were 49.0%, 31.7%, and 19.3%. None of the regressions achieved statistical significance (Table 4).

**Table 4:** Statistics for regressions between active contact location and WC. Slope, R^2^, and p values are reported for the model with only linear terms. “p for quadratic term” is the p-value for the quadratic term when added to the model. *n* = 81 for left STN, *n* = 82 for right STN

		slope (lbs/mm)	R^2^	p	p for quadratic term
left	X	−0.93	0.014	0.29	0.98
	Y	0.31	2.3 × 10^− 3^	0.67	0.93
	Z	−0.67	0.011	0.34	0.055
	PC1	−0.27	2.4 × 10^− 3^	0.66	0.13
	PC2	−1.0	0.023	0.18	0.12
	PC3	0.13	1.9 × 10^− 4^	0.90	0.017
right	X	1.3	0.027	0.14	0.70
	Y	0.054	7.5 × 10^− 5^	0.94	0.40
	Z	0.44	7.7 × 10^− 3^	0.43	0.085
	PC1	0.39	6.0 × 10^− 3^	0.49	0.094
	PC2	−0.31	2.4 × 10^− 3^	0.66	0.75
	PC3	1.4	0.029	0.12	1.0

## Discussion

This is the largest report of STN DBS effects on WC, providing further evidence that STN DBS causes weight gain in persons with PD. Our major finding was that active contact location with respect to the STN midpoint was not related to WC. WC was unlikely to be due to underweight patients achieving a healthy weight after surgery – only 4 patients were underweight at baseline, and 38% of patients with a healthy pre-operative weight became overweight or obese post-operatively. Among sex, disease duration, age, change in LED, change in MDS-UPDRS IV dyskinesia scores, and change in MDS-UPDRS III scores, only change in dyskinesia score was correlated with WC. This was only in the univariate (stepwise) model, and only accounted for 7.2% of the variance in WC. Change in LED significantly predicted WC when we excluded change in dyskinesia score, allowing us to include 11 additional patients. This is likely because change in LED and change in dyskinesia score are correlated. There was no significant difference in WC between patients who discontinued and those who did not discontinue dopamine agonists.

Our findings contrast with previous work suggesting that active electrode contact location is correlated with WC after STN DBS. Two studies found that medial contact location was associated with greater weight gain (Růžička et al. 2012; Steinhardt et al. 2023), but another found greater weight gain with lateral placement (Balestrino et al. 2017). A fourth study reported a cluster of active electrode contacts in the dorsal STN and adjacent regions outside the STN associated with WC (Eguchi et al. 2021). The conflicting results among prior studies could be related to methodological issues (e.g., how active sites were localized), differences in targeting between centers, or random variation due to small sample sizes (number of studies and number of subjects within each study), as summarized in Table 5.

**Table 5:** Summary of previous work relating stimulation location to WC

Study	Number of Participants	Localization Method	Effect observed
Růžička et al. 2012	20	X measured from wall of 3rd ventricle, Y and Z from midcommissural point	Weight gain greater for medial stimulation sites, R^2^ = 0.30, *p* < 0.01
Eguchi et al. 2021	14	Volume of Tissue Activated (VTA)	Weight gain associated with VTAs dorsally and outside STN bilaterally.
Balestrino et al. 2017	32	X measured from wall of 3rd ventricle, Y and Z from midcommissural point	Weight gain greater for lateral stimulation sites, R^2^ = 0.26, *p* < 0.01
Steinhardt et al. 2023	21	Volume of Tissue Activated (VTA)	Enhanced functional connectivity of limbic (ventromedial) STN correlated with weight gain

Several hypotheses for WC post-STN DBS have been advanced. Medial electrodes could activate limbic motivational STN regions or lateral hypothalamus to enhance food consumption (Růžička et al. 2012; Steinhardt et al. 2023). On the other hand, medial stimulation could inhibit orexigenic neurons in the lateral hypothalamic area, suppressing appetite (Balestrino et al. 2017). A third potential mechanism is increased synthesis of melanin concentrating hormone in zona incerta (ZI) due to stimulation in dorsal STN/ZI (Eguchi et al. 2021). However, the most straightforward explanation of our results and the prior contradictory literature is that active contact location makes at most a small contribution to WC after STN DBS. At 80% power to detect a type II error at α = 0.0083, this study was powered to detect an R^2^ of 0.022 for 82 subjects with 1 predictor (univariate position analysis), or 0.093 for 70 subjects with 6 predictors (multivariate analysis of clinical variables). That is, we could have missed an effect of clinical variables accounting for up to 9.3% of the variance in WC, or stimulation site accounting for up to 2.2% of the variance in WC due to our large sample size. These effects are smaller than those identified in previous studies (Table 5), and place an upper limit on the magnitude of WC effect that could be due to contact location, at least using our analytic approach in our cohort (Balestrino et al. 2017; Růžička et al. 2012). The span of the active electrode locations in our cohort was wide in all directions with similar variation compared to prior studies, suggesting that our findings are not an artifact of tightly clustered stimulation sites.

Most prior literature supports greater WC after STN DBS compared to GPi DBS in PD as well as in the limited literature evaluating change in weight in dystonia after DBS (Monteferrante et al. 2020; Mills et al. 2012; Eisinger et al. 2023; Locke et al. 2011). This suggests that even if there is no specific location within or around the STN implicated in WC, STN stimulation likely contributes to WC after DBS. Better understanding the differences in WC between STN DBS and GPi DBS may help elucidate nonmotor effects of stimulation at these two sites. LED reduction is more consistent with STN than GPi DBS (Follett et al. 2010). We found a borderline-significant correlation between LED reduction and WC after STN DBS, which has mixed support in the literature (Follett et al. 2010; Barichella et al. 2003; Gironell et al. 2002; Balestrino et al. 2017; Sauleau et al. 2009; Eguchi et al. 2021). This suggests that LED reduction is a marker of other factors driving WC, possibly fewer and less intense dyskinesias (also supported by our analysis). However, this seems unlikely to explain the difference in WC between STN and GPi DBS given their similar effects on dyskinesia reduction (Follett et al. 2010; Barichella et al. 2003; Gironell et al. 2002; Balestrino et al. 2017; Sauleau et al. 2009; Eguchi et al. 2021). Two studies looking at PET imaging in those with WC after STN and GPi DBS showed different areas in which 2-deoxy-2[18 F]fluoro-D-glucose uptake was associated with WC between these groups (Sauleau et al. 2014, 2016). While the STN DBS group showed more activation in limbic and associative areas, GPi showed activation of premotor areas as well as a trend towards less dyskinesia. It remains unclear why there is more weight gain with STN than GPi DBS.

The major implication of this study is that surgical teams should not adjust their target in an attempt to manipulate WC, whether to increase weight in underweight patients or decrease weight in overweight patients. While we cannot completely rule out a small effect of stimulation location on WC in our cohort, such an effect would be too small and inconsistent to risk suboptimal motor outcomes.

There are several limitations to this analysis (Table 6). Numerous psychosocial factors can contribute to WC that we did not account for, like changes in mood, cognition, impulse control disorders, or sleep. We also did not exclude patients based on change in medical history or exacerbation of other medical comorbidities, or follow changes to those comorbidities. We did not evaluate other physiologic parameters like hormonal levels, food intake, or metabolic measures of activity level and energy expenditure. Furthermore, we did not have the data to differentiate fat mass vs. fat-free mass. In terms of stimulation settings, we did not account for adjustments or amount of time on the settings recorded at follow-up. However, if any of these unmeasured variables were correlated with electrode position, that should bias our results away from the null hypothesis; if they are not correlated with electrode position, they should not affect the analysis.

**Table 6:** Limitations of the current study and potential biases

Limitation	Reason for Omission	Expected Bias
No control for intentional or disease-related weight change (e.g., dieting, intercurrent illness)	not systematically recorded in database	Confounding; WC may be incorrectly attributed to DBS rather than external health or behavioral factors
Absence of psychosocial, behavioral, and metabolic variables (e.g., diet, physical activity, mood, endocrine factors)	Not routinely captured in clinical dataset; difficult to quantify retrospectively	Residual confounding; may obscure or falsely attenuate associations between electrode location and weight change
No adjustment for disease progression or non-motor symptom changes	Longitudinal clinical detail on non-motor features not consistently available	Confounding; weight change may relate to disease evolution rather than stimulation location
Simplified electrode localization model (distance from midpoint in X, Y, Z only)	Analytical simplification; limitations of available imaging and modeling approaches	Misclassification bias; may dilute true spatial associations, particularly if effects are driven by small subregions rather than linear directional gradients.
Inability to model complex neuroanatomical relationships within and around the subthalamic nucleus	Structural complexity and proximity of neighboring nuclei may not be captured by simple coordinate-based approach	Reduced sensitivity to detect localized effects; potential false-negative findings regarding structure-function relationships
Retrospective study design	Use of existing clinical database rather than prospective protocol	Selection bias, information bias, and inconsistent data capture; limits causal inference

## Conclusion

In conclusion, WC after DBS is a poorly understood phenomenon. Active electrode contact location was not associated with WC after DBS in our cohort. While our analysis is limited by its retrospective nature, incomplete capture of potentially relevant confounders, and inability to rule out small effects, these data suggest that active contact locations within or around the STN are unlikely to significantly impact WC.

## Data Availability

The data that support the findings of this study are not openly available due to reasons of sensitivity and are available from the corresponding author upon reasonable request.
